# Supplementary material for: Predictability: A new distinguishing feature of cancer?
Source: PLoS One. 2024 Jun 12;19(6):e0305181. doi: 10.1371/journal.pone.0305181 (PMC11168650; doi:10.1371/journal.pone.0305181)
Supplement: Attachment — Submitted filename: Response predict.docx [file pone.0305181.s002.docx]

Emily Chenette	May 16, 2024 Editor-in-Chief PLoS One

Dear Editor

Thank you for considering the manuscript and for the reviewer's comment. Please find the enclosed response letter and the revised version.

We hope that you will find it interesting and suitable for publication in PLoS One.

Thanks

Ofer Gofrit

Ariel Aviv

**Reviewers' comments and responses:**

**Reviewer's Responses to Questions**

Comment 1: Is the manuscript technically sound, and do the data support the conclusions?

The manuscript must describe a technically sound piece of scientific research with data that supports the conclusions. Experiments must have been conducted rigorously, with appropriate controls, replication, and sample sizes. The conclusions must be drawn appropriately based on the data presented.

Reviewer #1: Yes

Reviewer #2: No

Response: All the raw data (obtained from the SEER program) is available in Supp 1.xlsx. Calculation of the predictability index (PI) is as follows: PI=median overall survival/standard error. Standard error= (upper 95%CI-lower 95%CI)/3.92 (page 4, materials and methods).

Comment 2: Has the statistical analysis been performed appropriately and rigorously?

Reviewer #1: Yes

Reviewer #2: No

Response: The study employs very simple statistics (only t-test comparing PI values of men and women).

Comment 3: Have the authors made all data underlying the findings in their manuscript fully available?

The [PLOS Data policy](http://www.plosone.org/static/policies.action#sharing) requires authors to make all data underlying the findings described in their manuscript fully available without restriction, with rare exception (please refer to the Data Availability Statement in the manuscript PDF file). The data should be provided as part of the manuscript or its supporting information, or deposited to a public repository. For example, in addition to summary statistics, the data points behind means, medians and variance measures should be available. If there are restrictions on publicly sharing data—e.g. participant privacy or use of data from a third party—those must be specified.

Reviewer #1: Yes

Reviewer #2: Yes

Response: None

Comment 4: Is the manuscript presented in an intelligible fashion and written in standard English?

PLOS ONE does not copyedit accepted manuscripts, so the language in submitted articles must be clear, correct, and unambiguous. Any typographical or grammatical errors should be corrected at revision, so please note any specific errors here.

Reviewer #1: Yes

Reviewer #2: Yes

Response: None

**Reviewer #1:** Thank you for the opportunity to review this interesting manuscript, which explores the variation in survival for different types of cancer. The study is original, and I am not aware of any similar undertaking. This line of research can highlight interesting clinical phenomena, and to inform both current clinical practice and future directions of research. I congratulate the authors on the idea to explore this interesting and provoking idea.

Major comment: While I appreciate the idea, I think the methods can be improved. Genetic heterogeneity is not the only cause of variability in clinical outcomes. The SEER dataset includes demographics, tumor subtypes and stage data- all directly associated with outcome variability. Why not include these data in the analysis? Perhaps some of the variability might be explained by heterogeneity in cancer subtypes/stage at diagnosis? This variation in outcomes will not necessarily represent variation in predictability.

Response: We wish to thank the reviewer for his kind words. The current manuscript is an initial attempt to turn the lights to predictability as a distinct feature of cancer. The proposed parameter "predictability index" is a preliminary attempt to quantify predictability. Adding more parameters, as the reviewer suggests, would certainly make it more accurate. We added these points to the discussion, however, the SEER program is not that accurate. It does not differentiate between cancer stages and does not consider comorbidity. We hope that future scientists will find interest in measuring cancer predictability and will come up with more accurate measures based on a more complete database as the reviewer suggests.

Minor comments
Comment 1: The manuscript can benefit from scientific editing by a native English speaker, for grammar and clarity.

Response: Any manuscript can be made better; but the current manuscript was revised by several native English speakers and by a professional scientific linguistic editor. Of course, any specific linguistic mistake can and should be corrected.

Comment 2: I’m not sure why the authors chose the subjective term “predictability” and not a more neutral “variation” in describing their results.

Response: Choosing an appropriate name was not an easy task. We wanted a term that will (quantitatively) reflect our ability to predict prognosis, therefore, we chose the term “predictability index”.

Comment 3: The authors should discuss the non-genetic factors which might influence patient outcome, which might be more variable in patients with some cancer types; various comorbidities, access to treatment issues etc. Genetics is a major factor, but not the only one.

Response: The important contribution of non-genetic factors to tumor's heterogeneity was added to the manuscript. These factors, especially epigenetic changes enable the development of genetically indistinguishable sub-populations with different sensitivity to systemic treatments. These changes can take the form of epigenetic heterogeneity (variability across cell population) that act as substrate for Darwinian selection or epigenetic plasticity (alterations in epigenetic state in response to external or internal stimuli with adaptive transcriptional, posttranslational, and metabolic plasticity) that act as a substrate for Lamarkian adaptation (Bell 2020, Inde 2018). Experimental models demonstrated that both transcriptional adaptation and posttranslational modifications can be found in acute myeloid leukemia, potentially increasing the variability of this disease and contributing to its low PI (Bell 2019, Irish 2004). These points were added to the "Discussion". Epigenetics in cancer is of course an extremely complex issue stretching far beyond what is mentioned here.

The issue of comorbidity and its potential contribution to patient variable prognosis was added to the "Discussion" (also in response to reviewer 2).

References

1. Bell CC, Gilan O. Principles and mechanisms of non-genetic resistance in cancer. Br J Cancer. 2020 Feb;122(4):465-472. doi: 10.1038/s41416-019-0648-6. Epub 2019 Dec 13. PMID: 31831859; PMCID: PMC7028722.
2. Inde Z, Dixon SJ. The impact of non-genetic heterogeneity on cancer cell death. Crit Rev Biochem Mol Biol. 2018 Feb;53(1):99-114. doi: 10.1080/10409238.2017.1412395. Epub 2017 Dec 18. PMID: 29250983; PMCID: PMC6089072.
3. Bell CC, Fennell KA, Chan YC, Rambow F, Yeung MM, Vassiliadis D, Lara L, Yeh P, Martelotto LG, Rogiers A, Kremer BE, Barbash O, Mohammad HP, Johanson TM, Burr ML, Dhar A, Karpinich N, Tian L, Tyler DS, MacPherson L, Shi J, Pinnawala N, Yew Fong C, Papenfuss AT, Grimmond SM, Dawson SJ, Allan RS, Kruger RG, Vakoc CR, Goode DL, Naik SH, Gilan O, Lam EYN, Marine JC, Prinjha RK, Dawson MA. Targeting enhancer switching overcomes non-genetic drug resistance in acute myeloid leukaemia. Nat Commun. 2019 Jun 20;10(1):2723. doi: 10.1038/s41467-019-10652-9. PMID: 31222014; PMCID: PMC6586637.
4. Irish JM, Hovland R, Krutzik PO, Perez OD, Bruserud Ø, Gjertsen BT, Nolan GP. Single cell profiling of potentiated phospho-protein networks in cancer cells. Cell. 2004 Jul 23;118(2):217-28. doi: 10.1016/j.cell.2004.06.028. PMID: 15260991.

**Reviewer #2:** The present article tries to prove that the survival of patients across different time points can be predicted and set a new carcinogenesis parameter

Comment 1: The drawbacks of such an assumption among other are:

1) Stage migration due to more sophisticated techniques during last decades.
   2) Different treatment efficacy during last decades.
   3) Different treatment toxicity during last decades.
   4) Different expected population survival due to the improvements in other specialties (e.g. cardiology) during last decades.

Response: Medicine keeps evolving and better diagnostic and therapeutic tools became available in more resent years. This led to stage migration, to more effective and less toxic therapies, and to better care of comorbidities. As the reviewer suggests, this phenomenon can decrease variability and improve the PI of more recently diagnosed patients. This important point was added to the discussion.
